# C-type lectin-like receptor 2: roles and drug target

**DOI:** 10.1186/s12959-024-00594-8

**Published:** 2024-03-19

**Authors:** Lan Sun, Zhe Wang, Zhiyan Liu, Guangyan Mu, Yimin Cui, Qian Xiang

**Affiliations:** 1 Department of Pharmacy, Peking University First Hospital, No. 6, Da Hong Luo Chang Street, Xicheng District, Beijing, 100034 China; 2 Department of Pharmacy Administration and Clinical Pharmacy, School of Pharmaceutical Sciences, Peking University Health Science Center, Beijing, China; 3 Institute of Clinical Pharmacology, Peking University, Beijing, China

**Keywords:** Platelets, C-type lectin-like receptor-2, Podoplanin, Thrombosis, Cancer

## Abstract

C-type lectin-like receptor-2 (CLEC-2) is a member of the C-type lectin superfamily of cell surface receptors. The first confirmed endogenous and exogenous ligands of CLEC-2 are podoplanin and rhodocytin, respectively. CLEC-2 is expressed on the surface of platelets, which participates in platelet activation and aggregation by binding with its ligands. CLEC-2 and its ligands are involved in pathophysiological processes, such as atherosclerosis, cancer, inflammatory thrombus status, maintenance of vascular wall integrity, and cancer-related thrombosis. In the last 5 years, different anti- podoplanin antibody types have been developed for the treatment of cancers, such as glioblastoma and lung cancer. New tests and new diagnostics targeting CLEC-2 are also discussed. CLEC-2 mediates thrombosis in various pathological states, but CLEC-2-specific deletion does not affect normal hemostasis, which would provide a new therapeutic tool for many thromboembolic diseases. The CLEC-2-podoplanin interaction is a target for cancer treatment. CLEC-2 may be applied in clinical practice and play a therapeutic role.

## Background

C-type lectin-like receptor 2 (CLEC-2) was first reported in 2000. CLEC-2 is a 32 kDa type II transmembrane protein that belongs to the C-type lectin superfamily receptor [1]. However, its expression in platelets was unknown until 2006 when Katsue Suzui-Inoue et al. described CLEC-2 as the first C-type lectin receptor found on platelets [2].

The most important ligands of CLEC-2 include two exogenous ligands rhodocytin [2] and the human immunodeficiency virus (HIV) [3] as well as an endogenous ligand, podoplanin (PDPN) [4].

The arachidonic acid (AA), adenosine diphosphate (ADP), and platelet-activating factor pathways are currently considered as the three classical pathways of platelet activation and aggregation. As for CLEC-2, it signals through phosphorylation of a single cytoplasmic YXXL sequence known as a hem-immunoreceptor tyrosine-based activation motif (hemITAM). CLEC-2 is dependent on the activation of spleen tyrosine kinase (Syk), leading to phosphorylation of downstream adapter proteins such as SH2 domain-containing leukocyte protein of 76 kDa (SLP-76) and phospholipase Cγ2 (PLCγ2), which increases calcium concentration and activates platelets [2, 5–7]. In humans, this process depends highly on the positive feedback exerted by platelet-derived ADP and thromboxane A2(TXA2) [8]. TXA2 enhances the phosphorylation of Syk and PLCγ2 [9]. Furthermore, CLEC-2 does not entirely depend on hemITAM signaling in hemostasis and thrombosis [10]. T-cell ubiquitin ligand-2 (TULA-2), a protein tyrosine phosphatase, negatively regulates CLEC-2 signaling. TULA-2 deficiency leads to the enhancement of the downstream Syk phosphorylation of CLEC-2 [11].

CLEC-2 has many other physiological and pathological functions in addition to participating in platelet activation. For instance, PDPN promotes tumor metastasis and progression by activating platelets after binding to CLEC-2 [12]. Based on the relationship between CLEC-2 and diseases, blocking the interaction between CLEC-2 and its ligands may be a new target for treating these diseases. Researchers have recently developed many anti-PDPN antibodies and extracted traditional Chinese medicine components to treat tumors and thrombosis. Their effects have been proven in animal experiments.

This review introduces the coding gene, expression in various cells, structure, ligand, physiological and pathological effects, and new drug research progress of CLEC-2. We mainly focus on new drug development.

## Brief introduction of CLEC-2

### Coding gene, expression, and structure

The gene encoding CLEC-2 is located on chromosome 12 within approximately 100 kb [1, 13]. CLEC-2 was first cloned from human bone marrow cells, and its cDNA was first reported to be selectively expressed on hepatocytes and some bone marrow-derived blood cells, such as monocytes, dendritic cells, and granulocytes [1]. Later, CLEC-2 was found to be highly expressed in platelets [2] and megakaryocytes [14]. A previous study has demonstrated that approximately 20% of phenotypical long-term hematopoietic stem cells (LT-HSCs) expressed CLEC-2. CLEC-2^high^ LT-HSCs were mainly involved in early megakaryopoiesis and served as a reserve for emergency thrombopoiesis under stress conditions [15]. However, CLEC-2 expression in human neutrophils has not been determined, and further studies are warranted to determine whether CLEC-2 exerts a regulatory effect on human polymorphonuclear leukocytes or other leukocytes [16].

As for the structure of CLEC-2, it is a 229-amino acid type II transmembrane receptor that is composed of an extracellular ligand-binding C-type lectin-like domain, stalk region, single transmembrane helix, and short cytoplasmic tail [17–18]. The tail contains a YXXL sequence, two conserved serine sequences at positions 21 and 27, and a partially conserved threonine sequence at position 9. The YXXL sequence is critical for CLEC-2 signaling [19]. CLEC-2 regulates Syk by forming dimers on the platelet surface. The two tandem SH2 domains possessed by Syk each bind to a single phosphorylated YXXL sequence on the cytoplasmic tail of CLEC-2. That is, CLEC-2 binds Syk in a 2:1 manner. CLEC-2 acts as a dimer to mediate platelet activation and aggregation [20].

### CLEC-2 and its ligands

#### Exogenous ligands

There are several types of exogenous ligands binding to CLEC-2. The first is the C-type lectin snake venom toxin, rhodocytin (also called aggretin), identified via chromatographic analysis [2]. Rhodocytin, a heterodimeric C-type lectin, binds to CLEC-2 to activate platelet signaling pathways [21]. Subsequently, diesel particles and sulfated polysaccharides, dextran sulfate and fucoidan, were discovered as exogenous ligands of CLEC-2 [21–22].

Katacine was a novel small-molecule exogenous ligand for CLEC-2 with a certain degree of specificity. It is a nonpolymeric proanthocyanidin extracted from the Polygonaceae family of flowering plants known as the knotweed family, which depends on Syk and Src to activate human platelets. This finding contributes to a better understanding of the role of CLEC-2 in human platelet activation and provides new ideas for further development of potential compounds with agonist effects on CLEC-2 [23]. Another recently identified exogenous ligand was galectin-9 (Gal-9). Gal-9, a family of carbohydrate-binding proteins with a broad range of immunomodulatory actions, has been reported to activate platelets. Gal-9 stimulated tyrosine phosphorylation of CLEC-2 and downstream proteins, inducing platelet activation [24].

#### Endogenous ligand

PDPN is the significant endogenous ligand of CLEC-2. PDPN is a type I transmembrane glycoprotein [25]. The extracellular domain of PDPN contains many serine and threonine residues as potential O-glycosylation sites. The binding of PDPN to CLEC-2 depends on the glycosylation of residues at the O-glycosylation site of PDPN, which plays a crucial role in platelet aggregation and tumor metastasis [4, 26]. In addition, PDPN binding to CLEC-2 is involved in venous thrombosis, inflammation in atherosclerosis, and wound repair. Furthermore, the interaction of CLEC-2 and PDPN promotes blood/lymphatic separation during embryonic development, maintains lymph node vascular integrity, and optimates adaptive immune response [27]. Other recently identified endogenous ligands are the smooth muscle calcium-binding protein S100A13 [28], hemin [29], and human Dectin-1 [30].

## The physiological and pathological effects of CLEC-2

### Physiological effects

CLEC-2 plays a central role in the formation of cerebrovascular [31] and blood/lymphatic vessel separation [32] during embryonic development. It interacts with PDPN expressed on neuroepithelial cells to induce platelet adhesion, aggregation, and secretion, which ultimately mediate the maturation and integrity of cerebrovascular and prevent hemorrhage [31]. CLEC-2 is involved in the maintenance of vascular integrity in inflammatory states. However, the mechanism of CLEC-2 activation is currently uncertain [33].

Current studies have demonstrated that CLEC-2 plays a vital role in liver regeneration. CLEC-2-associated liver regeneration was thought to be caused by the interaction between liver sinusoidal endothelial cells and platelets. CLEC-2 interacts with its endogenous ligand PDPN to induce IL-6 production, which ultimately promotes liver regeneration [34].

### Role of CLEC-2 in diseases

Some ligands have been demonstrated to interact with CLEC-2 to promote thrombus formation when the plaque is not ruptured. S100 calcium-binding protein A13 (S100A13) is expressed in the luminal surface of smooth muscle cells at the early stage of atherosclerosis or under certain pathological conditions. It activates platelets through CLEC-2 to promote thrombosis. CLEC-2 also binds to smooth muscle cells in normal arterial walls. However, normal arterial walls do not express PDPN or S100A13. There may be other, as yet undiscovered CLEC-2 ligands on normal vessel walls [28]. After plaque rupture in patients with advanced atherosclerosis, PDPN expressed in the plaque binds to CLEC-2 to induce platelet activation, thereby accelerating arterial thrombosis formation [35].

Binding of PDPN to CLEC-2 plays an important role in tumor growth and metastasis. PDPN is expressed in various cancer cells, such as ovarian cancer, hematologic tumors, glioblastoma, and osteosarcoma [36–39]. It also facilitates tumor metastasis by promoting an immunosuppressive microenvironment [40]. CLEC-2 bound to CpG oligodeoxynucleotides (CpG ODNs) resulted in platelet activation. CpG ODNs are short, single-stranded DNA molecules resembling bacterial DNA. CLEC-2 may be a target for adverse events in at-risk cancer patients treated with CpG ODNs [41]. Interestingly, the expression of CLEC-2 downregulated in gastric cancer [42] and liver cancer cells [43]. CLEC-2 exerts a protective effect on both cancers, suppressing cancer invasiveness and expressing epithelial-mesenchymal transition. In conclusion, CLEC-2 plays different roles in different cancers.

CLEC-2 plays a crucial role in vascular inflammation. The interaction of PDPN and CLEC-2 not only contributes to the formation of sepsis-related immune thrombosis [44] but also restricts further deterioration of sepsis by controlling cytokine storm [45], regulating immune cell infiltration [46] and inducing the release of complement inhibitors [47]. Interestingly, CLEC-2 is involved in coronavirus disease 2019 (COVID-19)-related thrombosis. Platelets release von Willebrand factor (vWF) and platelet factor 4 (PF4) from α granules and express P-selectin and CLEC-2. Endothelial cell injury releases VWF and factor VIII. These changes collectively contribute to developing COVID-19-related thrombosis [48]. Deep vein thrombosis (DVT) is a thromboinflammatory disorder developing largely as sterile inflammation in the vessel wall. Payne et al. found that CLEC-2 interacted with PDPN, which was upregulated in the vein wall, to promote thrombosis [49].

CLEC-2 is also involved in tumor-associated thrombosis. Shirai et al. found CLEC-2 interacted with PDPN to promote thrombus formation in tumor blood vessels and indirectly induced tumor cell proliferation in melanoma [36]. High PDPN expression in cancer cells induced platelet aggregation and was associated with an increased risk of venous thromboembolism (VTE) and poor prognosis [50–51].

The binding of PDPN to CLEC-2 was found to inhibit T cells by using a mouse model of experimental autoimmune encephalomyelitis. PDPN on T cells was overexpressed on Th17 cells to control inflammation. PDPN was a marker of nonpathogenic Th17 [52–53]. In rheumatoid arthritis, PDPN was overexpressed in Th17 cells and fibroblasts, which significantly increased IL-17 secretion and induced inflammation [54]. The results indicated that PDPN was a marker of pathogenic Th17, mainly depending on the type of inflammation.

**Fig. 1:**
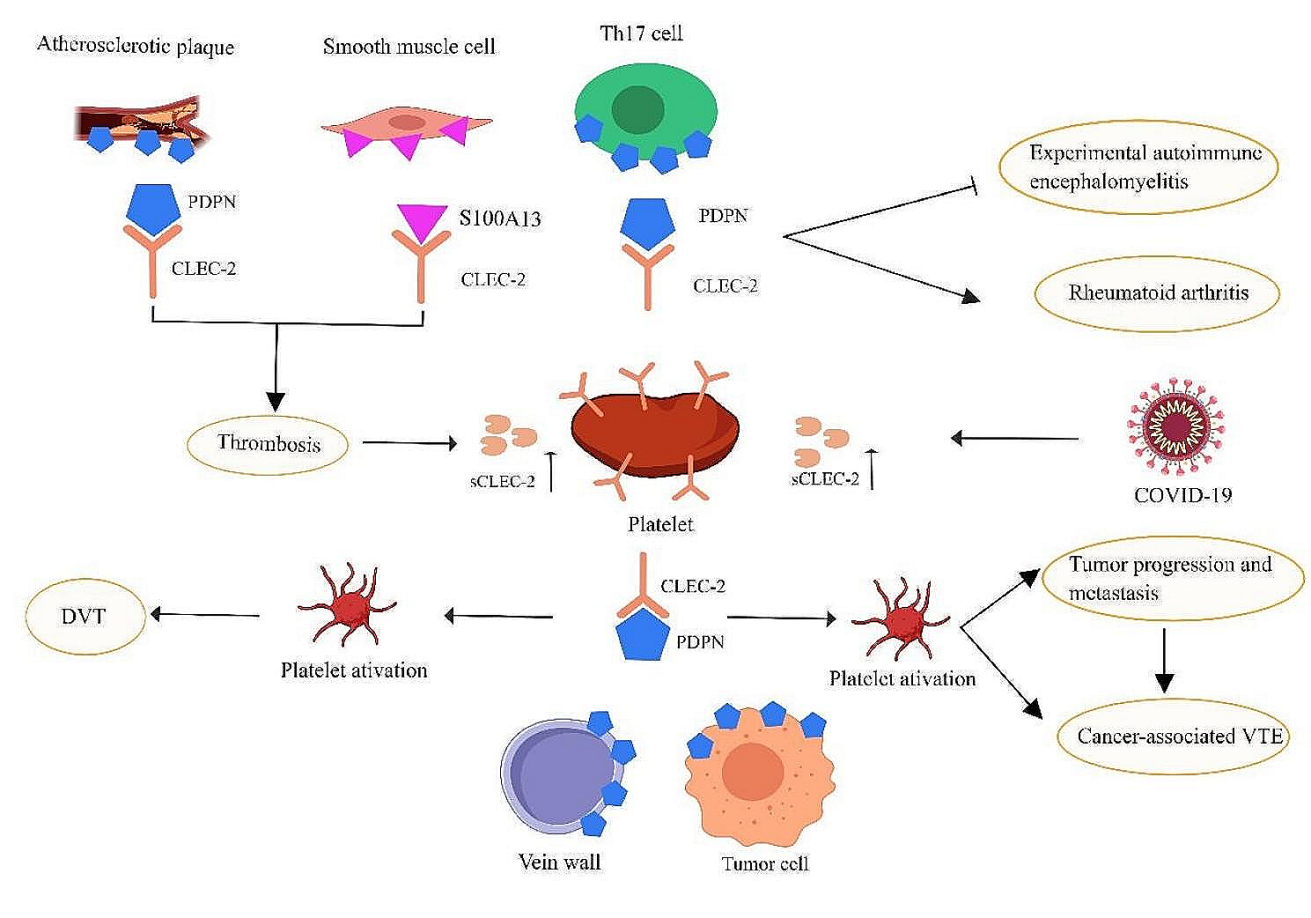
Role of CLEC-2 in main diseases

The relationship between CLEC-2 and major diseases is summarized in Fig. 1.

CLEC-2 expressed on the platelet surface interacts with PDPN expressed on different cell surfaces to mediate diseases such as tumor progression and metastasis, tumor-associated thrombosis, venous thrombosis, arterial thrombosis, and immune diseases. Furthermore, sCLEC-2 levels are elevated in patients with COVID-19 and coronary artery disease.

Created with MedPeer (www.medpeer.cn).

## CLEC-2, as a target in drug research

### New drugs

In this subsection, we will mainly discuss research on new drugs. However, new drugs are still being developed in preclinical studies. We expect to enter the clinical stage for the following new compounds or older drugs with new indications in the future. The preclinical studies are presented in Table 1 at the end of the article.

**Table 1:** The mechanism of new reagents

Disease	Reagent	Mechanism
Tumor Metastasis and Thrombosis	NZ-12 [55]	Human-mouse chimeric antibody treating brain cancer and lung cancer.
	ChLpMab-23 [56]	Human-mouse chimeric antibody showed high sensitivity to glioblastoma and oral cancer.
	ChLpMab-2 [57]	Human-mouse chimeric antibody showed high sensitivity to glioblastoma, mesothelioma, and lung cancer.
	LpMab-21 [58]	High ADCC and CDC activity against ovarian cancer, glioblastoma, and lung cancer.
	LpMab-23 [59]	Anti-PDPN to treat oral cancer.
	NZ-27, P1027 [60]	High CDC activity against glioblastoma.
	P2-0, MS-1, MS-3, and MS-4 [61]	Inhibitors of the binding of PDPN and CLEC-2 suppressed the growth of the tumor.
	PG4D2, AP201 [62]	Against the interaction of PDPN and CLEC-2, both of which had inhibitory effects on the growth and metastasis of osteosarcoma.
	LpMab-7 [63]	Diagnostic tool to identify patients with PDPN-positive osteosarcoma.
	2A2B10 [64]	Inhibition of hematogenous metastasis and thrombosis of PDPN-positive melanoma without a significant bleeding tendency.
	2CP [65]	Anticancer metastatic activity *in vivo.*
	Co-HP [67]	The binding of Co-HP to CLEC-2 blocked the interaction between CLEC-2 and PDPN.
	Fucoidan [68]	Restrained the progression of gastric cancer by up-regulating the level of CLEC-2.
	AAWAP [69]	Irreversibly blocked the effects of PDPN and CLEC-2 in tumors.
	Bisdemethocycurcumin and demethoxycurcumin [70]	Natural antagonists of CLEC-2 with anti-colon cancer activity.
Atherosclerosis	Ibrutinib [71]	Btk is located in the downstream of Syk, blocking CLEC-2-mediated platelet activation and tyrosine phosphorylation.
	MK-1026 [73]	
	TRPM7 kinase [74]	The deletion of TRPM7 inhibited hemITAM-PLCγ2-mediated intracellular Ca^2+^ mobilization.
Wound Healing	Dasatinib [76]	Inhibited the downstream signaling molecules Src and Syk of CLEC-2 and blocked CLEC-2-mediated platelet activation.

#### Tumor metastasis and thrombosis

##### Anti-PDPN and Anti-CLEC-2 antibodies

As for anti-PDPN antibodies, there can be divided into human–mouse chimeric antibodies, monoclonal antibodies, and neutralizing antibodies. The human–mouse chimeric antibodies, NZ-12 [55], chLpMab-23 [56], and chLpMab-2 [57] have been developed. All these antibodies showed high response to cancer. For example, NZ-12, the constant regions of which consist of IgG1 heavy chain and lambda light chain. NZ-12 significantly increased antibody-dependent cellular cytotoxicity (ADCC) and complement-dependent cytotoxicity (CDC) activities against glioblastoma and lung cancer. NZ-12 may be used for the treatment of PDPN-expressing brain cancer and lung cancer [55]. In terms of monoclonal antibodies, researchers developed LpMab-21 [58], LpMab-23 [59], P1027, NZ-12f [60], P2-0, MS-1, MS-3, and MS-4 [61]. For instance, one patent discovers a novel domain of PDPN, the PLAG4 domain, which is important for PDPN binding to CLEC-2. The anti-PDPN monoclonal antibodies recognizing this region are P2-0, MS-1, MS-3, and MS-4. These antibodies suppressed the growth of the tumor [61]. Takemoto et al. developed neutralizing antibodies PG4D2 and AP201 against the interaction of PDPN and CLEC-2, both of which exerted inhibitory effects on the growth and metastasis of osteosarcoma. These antibodies are a new therapeutic strategy for PDPN-positive osteosarcoma [62].

Moreover, some anti-PDPN monoclonal antibodies identified patients with PDPN-positive tumors. A novel anti-PDPN monoclonal antibody, LpMab-7, had high sensitivity to detect metastatic lesions of osteosarcomas. It may be helpful for molecular targeting therapy for osteosarcomas [63].

Many studies on anti-PDPN antibodies have been conducted, but only one novel antibody targeting CLEC-2 has been reported. Anti-CLEC-2 monoclonal antibody 2A2B10 has been demonstrated to inhibit hematogenous metastasis and thrombosis of PDPN-positive mouse melanoma without a significant bleeding tendency [64].

Overall, existing studies have demonstrated that anti-PDPN antibodies can be developed for clinical use. In the future, finding the downstream pathways of PDPN leading to tumor metastasis will be crucial for developing effective drugs against PDPN. Attention should be paid to potential adverse reactions when developing new anti-PDPN antibodies as PDPN is also expressed in normal tissues. Interestingly, LpMab monoclonal antibody series have been reported to specifically recognize aberrantly glycosylated PDPN in cancer cells, thereby avoiding adverse reactions [65]. While such antibodies are promising to avoid side effects, they may not recognize PDPN upregulation in endothelial cells and monocytes during chronic inflammation. In other words, such antibodies may not prevent the adverse effects of cancer-associated thrombosis.

##### Small-molecule compounds

Chang et al. developed 2CP, the first platelet antagonist with CLEC-2 binding activity. 2CP has anticancer metastatic activity in vivo. It enhances the therapeutic effect of cisplatin in experimental animal models without causing bleeding risk [66].

Another small-molecule compound is cobalt hematoporphyrin (Co-HP). Tsukiji et al. obtained Co-HP through compound screening and modification, which bound to the N120 and K211 sites of CLEC-2. Co-HP at a concentration of 1.53 µM inhibited platelet aggregation mediated through CLEC-2 but not that mediated through other receptors. Co-HP binding to CLEC-2 blocked the interaction between CLEC-2 and PDPN in lung cancer. Co-HP inhibited lung cancer metastasis and arterial or venous thrombosis in vivo without increasing bleeding risk [67]. However, its low affinity, oral availability, and toxicity indicate that it is unsuitable for clinical development.

##### Macromolecular compounds

As mentioned above, the expression of CLEC-2 is downregulated in gastric cancer. As an exogenous ligand of CELC-2, Fucoidan is a sulfated polysaccharide extracted from the cell wall matrix of various brown seaweed, affecting many pathophysiological processes, including cancer. Fucoidan inhibited the progression of gastric cancer by upregulating the CLEC-2 level in gastric cancer cells through the transcription factor caudal type homeobox transcription factor 2, an important regulator of gut homeostasis [68]. Nonetheless, clinical trials are need to be conducted to prove its effectiveness.

##### Traditional Chinese medicine

As a treasure of the Chinese nation, traditional Chinese medicine has also been found to play a significant role in treating tumors by blocking the effects of CLEC-2 and PDPN. Tseng et al. showed that a polysaccharide-containing fraction (AAWAP) from the water extract of Artemisia argyi leaves irreversibly blocked the effects of PDPN and CLEC-2 in tumors in a dose-dependent manner. Notably, AAWAP is nontoxic to cells and platelets [69]. AAWAP may effectively inhibit tumor metastasis and become a potential drug for cancer treatment. Another study found that bisdemethocycurcumin and demethoxycurcumin were natural antagonists of CLEC-2 with anti-colon cancer activity through compound screening. Further studies are warranted to confirm this result in vivo and in vitro [70].

Traditional Chinese medicine has the advantages of safety, efficacy, and low toxicity. Further research on the components of Chinese medicine may also be an effective way to discover new drugs.

#### Atherosclerosis

Antiplatelet therapy has long been the foundation of atherosclerotic event treatment and prevention. Studies have demonstrated that Bruton’s tyrosine kinase (Btk) is located in the downstream of Syk. Btk inhibitors can be used as novel antiplatelet agents in the treatment of thrombotic diseases. The low-dose Btk inhibitor ibrutinib blocked CLEC-2-mediated platelet activation and tyrosine phosphorylation. Ibrutinib is an irreversible Btk inhibitor that increases bleeding risk [71]. This impact of ibrutinib is likely due to off-target effects on kinases such as Src family kinases, which are supported by different effects of the CLEC-2 signaling pathway at different concentrations of Ibrutinib [72]. Other specific Btk inhibitors do not have these off-target effects, which aligns with reducing bleeding side effects in patients. A recent study developed MK-1026, a reversible Btk inhibitor. It inhibited CLEC-2-mediated platelet aggregation and reduced the risk of bleeding in patients. It is also a powerful drug for treating thrombotic diseases [73].

Transient receptor potential melastatin 7 (TRPM7) kinase is a bifunctional protein that forms a constitutively active Mg^2+^ and Ca^2+^ permeable channel. The study found that TRPM7 deletion inhibited hemITAM-PLCγ2-mediated intracellular Ca^2+^ mobilization using a mouse model with TRPM7 kinase activity deletion. In other words, TRPM7 kinase plays a key role in CLEC-2-induced platelet activation. Furthermore, disruption of TRPM7 kinase activity did not result in intracranial hemorrhage in mice after ischemic stroke. These results suggest that TRPM7 kinase is a safety potential target for anti-arterial thrombosis [74].

#### Wound healing

Depletion of CLEC-2 has been proposed as a potential novel mechanism to promote skin wound healing. This phenomenon increased fibrin(ogen) deposition, reduced inflammation, and promoted angiogenesis [75]. Targeting CLEC-2 at the wound site may be a new approach to promote wound healing.

There is already a preclinical study on wound healing. Dasatinib, a pan-tyrosine kinase inhibitor, is currently used to treat chronic myeloid leukemia. Recently, Wichaiyo et al. found that dasatinib promoted skin wound healing [76]. Dasatinib inhibited tyrosine kinases Src and Syk, which are the downstream signaling molecules of CLEC-2. Therefore, dasatinib blocked CLEC-2-mediated platelet activation, leading to self-limited inflammatory bleeding and fibrinogen/fibrin deposition, associated with reduced inflammation, increased re-epithelialization, and enhanced angiogenesis.

### New tests and diagnostics

Brown et al. generated a humanized CLEC-2 (hCLEC-2^KI^) mouse model by replacing mouse genes with human variants using CRISPR technology. In this mouse model, hCLEC-2 can be immunodepleted, addressing the limitation that the in vivo role of hCLEC-2 is not easy to study experimentally in humans. The hCLEC-2^KI^ mouse model provides a theoretical basis for studying anti-hCLEC-2 agents in vivo [77]. Furthermore, Watanabe et al. developed a drug screening system based on the combination of a pull-down assay using a centrifugal filter unit and a slot blot assay. An immunoglobulin Fc domain-CLEC-2 fusion protein was used as a bait to capture PDPN derived from PDPN-overexpressing HeLa cells in the presence and absence of the test compound. The system effectively screened out small-molecule inhibitors for cancer and thrombosis treatment by inhibiting the PDPN–CLEC-2 interaction [78].

In recent years, an increasing number of studies have confirmed the clinical significance of CLEC-2 in cardiovascular and cerebrovascular diseases. Platelet activation releases soluble forms of platelet membrane proteins, such as soluble CLEC-2 (sCLEC-2). sCLEC-2 binds to the ligands of CLEC-2 and inhibits membrane-bound CLEC-2 activation. Fei et al. conducted a multicenter, cross-sectional study of 216 patients, including 14 cases of stable angina pectoris (SAP, non-ACS) and 202 cases of acute coronary syndrome (ACS). The results showed that the plasma levels of sCLEC-2 were significantly increased in patients with SAP (133.67 (88.76-220.09) pg/mL) and ACS (134.16 (88.88-225.81) pg/mL) compared with control groups (65.69 (55.36-143.22) pg/mL) [79]. A prospective control study involving 352 acute ischemic stroke (AIS) patients and 112 healthy controls was conducted. Increased plasma CLEC-2 (pCLEC-2) levels were associated with worse stroke (odds ratio (OR) = 1.97, 95% confidence interval (CI) = 1.11–3.50, *p* = 0.02) and poor prognosis (OR = 1.70, 95% CI = 1.17–2.48, *p* = 0.006) [80]. Another prospective study followed 352 AIS patients for one year to evaluate the impact of pCLEC-2 level on the long-term prognosis of these patients. These patients with high pCLEC-2 had an increased risk of death and combined endpoints [81].

Interestingly, sCLEC-2 levels help predict the prognosis of COVID-19. Wada et al. compared the sCLEC-2 levels of patients with severe acute respiratory syndrome coronavirus 2 (SARS-CoV-2) infection (*n* = 46) with those of patients with other infections (*n* = 127). The plasma sCLEC-2 levels in patients with COVID-19 were significantly higher than those in patients with other infections (*p* < 0.001). sCLEC-2 levels increased with the severity of COVID-19 [82].

Recently, a patent provided a means of easy diagnosis using a blood test in medical care for hemorrhagic stroke. This method involved measuring the concentration of sCLEC-2 (or a value calculated by dividing sCLEC-2 concentration by platelet count) present in blood samples obtained from the patients suspected to have or diagnosed with hemorrhagic stroke [83]. The patent showed that the sCLEC-2/platelet count ratio (C2PAC index) is also crucial for diagnosing the disease, including the early evaluation of coagulation function in patients with sepsis and postoperative VTE.

In a new clinical study, 70 patients with sepsis were divided into the sepsis-induced disseminated intravascular coagulation (SID) group (*n* = 44) and the non-SID group (*n* = 26), and the C2PAC index was compared between the two groups. The C2PAC index of the SID group was significantly higher than that of the non-SID group (*p* < 0.001). The C2PAC index may be useful for the early evaluation of coagulation function in patients with sepsis [84]. Another clinical trial from Japan enrolled 44 patients with high-grade gliomas, which divided them into isocitrate dehydrogenase (IDH) wild-type and IDH mutant groups [85]. The results indicated that the mean levels of the sCLEC-2 (*p* = 0.0004) and C2PAC (*p* = 0.0002) indices in patients with IDH wild-type were significantly higher than those in patients with IDH mutation. In the IDH wild-type group, the C2PAC index in the VTE group was significantly higher than that in the non-VTE group (*p* = 0.0492). The C2PAC index is a potential indicator for detecting postoperative VTE formation. We have summarized the main contents of new tests and diagnostics in Table 2.

**Table 2:** The characteristics of new tests and diagnostics

Model/Index	Disease/Application
Humanized CLEC-2 mouse model	Screened out small-molecule inhibitors [77]
Drug screening system	Screened out small-molecule inhibitors [78]
sCLEC-2/ pCLEC-2↑	Coronary artery disease [79]
	Acute ischemic stroke [80–81]
	COVID-19 [82]
C2PAC↑	Coagulation function in patients with sepsis [83]
	Postoperative VTE formation [85]

sCLEC-2, soluble CLEC-2; pCLEC-2, plasma CLEC-2; C2PAC, sCLEC-2/platelet count ratio; COVID-19, coronavirus Disease 2019; VTE, venous thromboembolism

### Challenge and approaches to identifying novel drugs against CLEC-2 and PDPN

Current antiplatelet therapy carries a risk of excessive bleeding. Therefore, a new antiplatelet agent that prevents thrombosis without causing bleeding needs to be developed. CLEC-2 has been proposed as such a target. CLEC-2 is a physiological target protein of PDPN, implying that it is involved in PDPN-induced platelet aggregation, tumor metastasis, and other cellular responses related to PDPN [86]. The hemostatic effect of CLEC-2 in humans is negligible [87]. Thus, targeting CLEC-2 may have an advantage.

Although various anti-PDPN antibodies have been developed, validation using tumor cells that endogenously express PDPPN remains limited. PDPN is expressed in a variety of tumors. However, few PDPN-expressing cell lines are obtained from public cell banks. Furthermore, few of the cell lines obtained are suitable for in *vivo* metastasis models, which may be due to changes in the properties of specific tumor cells during the establishment of cell lines [65].

At present, Takemoto et al. established and successfully generated PDPN-positive lung SCC patient-derived cell lines, but further analysis using patient-derived models needs to be conducted [65]. More studies on PDPN-positive tumor cells and tumor/metastasis models containing patient-derived models, such as searching for the downstream pathways of PDPN leading to tumor metastasis, are warranted to provide insights into the development of novel therapies targeting the interaction of PDPN and CLEC-2.

Because antibodies or biologics are not orally available, they are not ideal for long-term prophylaxis in patients at risk for thrombosis. Therefore, there is an urgent need to develop small-molecule targeted drugs. However, interactions between protein ligands and receptors are challenging to model in small molecules as the sites of interaction may have relatively few features and cover a large surface area [88].

Because CLEC-2 does not have a high-affinity ligand, the structure-based drug design is more appropriate than the ligand-based drug design. Virtual studies, HTS studies, and molecular dynamic simulations can be selected [88].

Therefore, drugs targeting CLEC-2 are expected to be antithrombotic, antiplatelet, and antimetastatic agents with minimal bleeding side effects. However, no drug targeting CLEC-2 has been found to enter clinical trials. Future studies are expected to develop platelet aggregation inhibitors with few bleeding side effects and antimetastatic drugs that inhibit cancer metastasis, as well as their candidate compounds.

## Conclusion

CLEC-2 mediates thrombosis in various pathological states, but CLEC-2-specific deletion does not affect normal hemostasis, which would provide a new therapeutic tool for many thromboembolic diseases. The interaction of PDPN and CLEC-2 is a target for cancer treatment. Due to the multiple roles of CLEC-2 in regulating platelet function during disease progression, its targeting in disease must be closely monitored.

The latest drug research focuses on anti-PDPN antibodies for the treatment of various cancer types. Only one novel antibody targeting CLEC-2 has been reported. CLEC-2-binding small molecules need to be developed further. In addition, the new drug research requires clinical trials to verify its clinical significance.

Current studies had limitations. First, most current studies are animal models and clinical studies still need to be completed. Additionally, the CLEC-2 expression differs between mice and humans. In humans, CLEC-2 is only found in platelets, megakaryocytes, and the liver. In mice, CLEC-2 is also expressed in several immune cells, including dendritic cells, peripheral neutrophils, inflammatory macrophages, and B cells. Last but not least, mouse platelets express only GPVI and CLEC-2 as receptors for the ITAM pathway, whereas human platelets also contain the human immunoglobulin GFc segment II receptor (FcγRIIA) protein. Thus, the findings from mouse experiments may not be applicable to humans. In the future, further studies can be conducted from the above three aspects or through other mechanisms of action so that CLEC-2 can be more effectively applied in clinical practice and play a better therapeutic role.

## Data Availability

No datasets were generated or analysed during the current study.

## Abbreviations

CLEC-2: C-type lectin-like receptor-2
HIV: human immunodeficiency virus
PDPN: podoplanin
AA: arachidonic acid
ADP: adenosine diphosphate
hemITAM: hem-immunoreceptor tyrosine-based activation motif
Syk: spleen tyrosine kinase
SLP-76: SH2 domain-containing leukocyte protein of 76 kDa
PLCγ2: phospholipase Cγ2
TXA2: thromboxane A2
Btk: Bruton’s tyrosine kinase
TULA-2: T-cell ubiquitin ligand-2
LT-HSCs: long-term hematopoietic stem cells
HTS: high-throughput screening
Gal-9: galectin-9
S100A13: S100 calcium-binding protein A13
CpG ODNs: CpG oligodeoxynucleotides
COVID-19: coronavirus disease 2019
vWF: von Willebrand factor
PF4: platelet factor 4
DVT: deep vein thrombosis
VTE: venous thromboembolism
ADCC: antibody-dependent cellular cytotoxicity
CDC: complement-dependent cytotoxicity
Co-HP: cobalt hematoporphyrin
AAWAP: polysaccharide-containing fraction
Btk: Bruton’s tyrosine kinase
GPVI: glycoprotein VI
TRPM7: transient receptor potential melastatin 7
hCLEC1-2^KI^: human CLEC-2
pCLEC-2: plasma CLEC-2
CAD: coronary artery disease
AIS: acute ischemic stroke
ACS: acute coronary syndromes
OR: odds ratio
CI: confidence interval
SARS-CoV-2: severe acute respiratory syndrome coronavirus 2
C2PAC index: sCLEC-2/platelet count ratio
SID: sepsis-induced disseminated
IDH: isocitrate dehydrogenase
